# Complete Genome Sequence of Stenotrophomonas maltophilia Myophage Marzo

**DOI:** 10.1128/mra.01202-21

**Published:** 2022-02-28

**Authors:** Janki Patel, Brenda Godoy, James Clark, Ben Burrowes, Ry Young, Mei Liu

**Affiliations:** a Department of Biochemistry and Biophysics, Texas A&M University, College Station, Texas, USA; b Center for Phage Technology, Texas A&M University, College Station, Texas, USA; DOE Joint Genome Institute

## Abstract

Stenotrophomonas maltophilia is a Gram-negative opportunistic bacterium that is increasingly being associated with infections. Here, we report the complete genome of the S. maltophilia myophage Marzo, with a 159,384-bp genome encoding 268 proteins, 23 tRNAs, and 1 transfer-messenger RNA. Marzo is closely related to S. maltophilia phages IME-SM1 and Mendera.

## ANNOUNCEMENT

Stenotrophomonas maltophilia is found in aqueous habitats, including plant rhizospheres and animals, and is an opportunistic Gram-negative bacterium that can cause infections in tissues ranging from the skin to the heart in immunocompromised individuals (1). We are interested in studying S. maltophilia phage genomes in the interest of exploring potential therapeutic treatment options.

Phage Marzo was isolated from an activated sludge sample collected from the Texas A&M wastewater treatment plant in September 2019, using the soft agar overlay method (2) with S. maltophilia (ATCC 17807) as the propagation host grown aerobically at 30°C in nutrient broth or agar (BD). Marzo DNA was purified from ∼8 mL phage lysate using the Promega Wizard DNA cleanup system, as described previously (3). Sequencing libraries were prepared as 300-bp inserts using a Swift 2S Turbo kit and sequenced on an Illumina MiSeq system with paired-end 150-bp reads using 300-cycle v2 chemistry. The 106,506 total sequence reads were quality controlled using FastQC (www.bioinformatics.babraham.ac.uk/projects/fastqc), trimmed with FASTX-Toolkit v0.0.14 (http://hannonlab.cshl.edu/fastx_toolkit), and assembled using SPAdes v3.5.0 (4). A raw contig of 159,439 bp was obtained, and its end sequences were manually corrected with Sanger sequencing of a PCR product amplified from the contig ends (forward primer, TGAACTTCTCCAGCCCGAAC; reverse primer, TGTAGCGAGCCCTGATCTCT). PhageTerm was used to predict phage termini from the raw sequencing reads (5). Phage Marzo was annotated using the Center for Phage Technology (CPT) Galaxy-Apollo phage annotation platform (https://cpt.tamu.edu/galaxy-pub) (6–8). Gene calling included GLIMMER v3.0 (9) and MetaGeneAnnotator v1.0 (10). tRNA and transfer-messenger RNA (tmRNA) genes were detected using ARAGORN v2.36 (11) and tRNAscan-SE v2.0 (12). Gene function was predicted using InterProScan v5.48 (13), BLAST v2.9.0 (14) with the NCBI nonredundant and Swiss-Prot databases (15), TMHMM v2.0 (16) for transmembrane domains, HHPred (17), LipoP v1.0 (18) for lipoproteins, and SignalP v5.0 (19). Genome-wide DNA sequence similarity to top BLAST nucleotide hits (from the NCBI nucleotide database) was calculated by progressiveMauve v2.4 (20). All tools were run with default settings unless otherwise specified.

**FIG 1:**
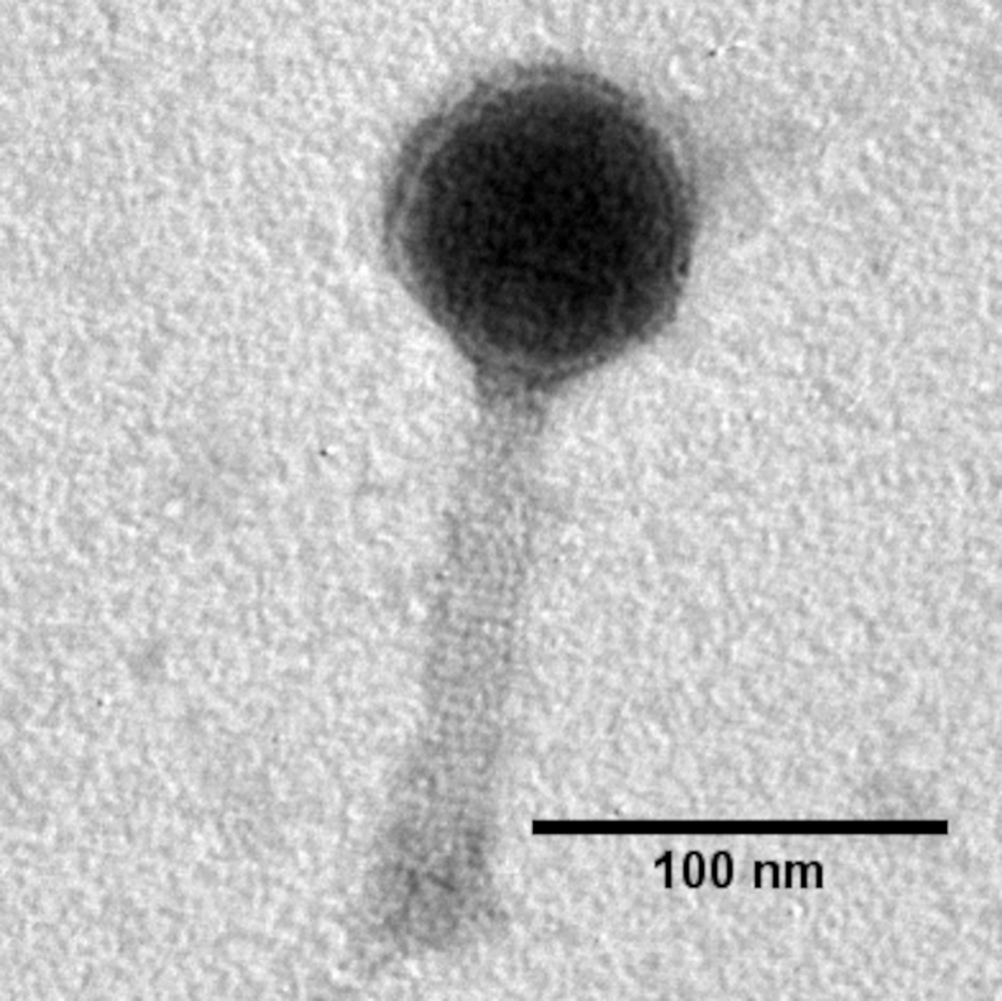
TEM of phage Marzo. Phage particles were diluted with TEM buffer (20 mM NaCl, 10 mM Tris-HCl [pH 7.5], 2 mM MgSO_4_) and captured on freshly glow-discharged, Formvar carbon-coated grids. The grids were stained with 2% (wt/vol) uranyl acetate and observed on a JEOL 1200 EX TEM at 100-kV accelerating voltage at the Microscopy and Imaging Center at Texas A&M University.

Phage Marzo was determined to be a myophage via negative staining with 2% (wt/vol) uranyl acetate and imaging by transmission electron microscopy (TEM) at the Texas A&M University Microscopy and Imaging Center (Fig. 1). The completed 159,384-bp myophage Marzo genome has 24-fold sequencing coverage and a G+C content of 54%. PhageTerm was unable to predict phage termini from the raw sequencing reads, but due to its similarities in terms of morphology and genome size to the canonical phage T4, Marzo likely uses headful packaging. Twenty-three tRNA genes, 1 tmRNA gene, and 268 protein-coding genes were found, with a coding density of 93%. The tRNA genes were found in two clusters, one with 3 tRNAs and the tmRNA and the other with 20 tRNAs. The Marzo tmRNA was highly similar to the SsrA *Betaproteobacter*-class tmRNA of S. maltophilia, as determined by sequence analysis at RNAcentral (21). Comparative genomics revealed that Marzo has ≥92% nucleotide identity to other S. maltophilia myophages, namely, IME-SM1 (GenBank accession number KR560069), YB07 (GenBank accession number NC_048755), and Mendera (GenBank accession number NC_048804). Some structural genes could be identified, encoding tail completion scaffold, portal, major capsid, baseplate wedge, and tail tube proteins. In addition, although no holin or endolysin genes could be identified, two spanin gene pairs were identified, one of the overlapping class and the other of the embedded class.

### Data availability.

The Marzo genome was deposited in GenBank with accession number MZ326868. The associated BioProject, SRA, and BioSample accession numbers are PRJNA222858, SRR14095257, and SAMN18509700, respectively.
